# Reinforcement learning for adaptive threshold control of restorative brain-computer interfaces: a Bayesian simulation

**DOI:** 10.3389/fnins.2015.00036

**Published:** 2015-02-12

**Authors:** Robert Bauer, Alireza Gharabaghi

**Affiliations:** ^1^Division of Functional and Restorative Neurosurgery and Division of Translational Neurosurgery, Department of Neurosurgery, Eberhard Karls University TuebingenTuebingen, Germany; ^2^Neuroprosthetics Research Group, Werner Reichardt Centre for Integrative Neuroscience, Eberhard Karls University TuebingenTuebingen, Germany

**Keywords:** reinforcement learning, classification accuracy, neurofeedback, functional restoration, neurorehabilitation, brain-computer interface, brain-machine interface, brain-robot interface

## Abstract

Restorative brain-computer interfaces (BCI) are increasingly used to provide feedback of neuronal states in a bid to normalize pathological brain activity and achieve behavioral gains. However, patients and healthy subjects alike often show a large variability, or even inability, of brain self-regulation for BCI control, known as BCI illiteracy. Although current co-adaptive algorithms are powerful for *assistive* BCIs, their inherent class switching clashes with the operant conditioning goal of *restorative* BCIs. Moreover, due to the treatment rationale, the classifier of restorative BCIs usually has a constrained feature space, thus limiting the possibility of classifier adaptation. In this context, we applied a Bayesian model of neurofeedback and reinforcement learning for different threshold selection strategies to study the impact of threshold adaptation of a linear classifier on optimizing restorative BCIs. For each feedback iteration, we first determined the thresholds that result in minimal action entropy and maximal instructional efficiency. We then used the resulting vector for the simulation of continuous threshold adaptation. We could thus show that threshold adaptation can improve reinforcement learning, particularly in cases of BCI illiteracy. Finally, on the basis of information-theory, we provided an explanation for the achieved benefits of adaptive threshold setting.

## Introduction

Restorative brain-computer and brain-machine interfaces (BCI/BMI)—emerging rehabilitation technologies for neurofeedback training—seek to reduce disease-specific symptoms in a variety of brain disorders (Wyckoff and Birbaumer, 2014). Unlike classical *assistive* BCIs, whose goal is to replace lost functions by controlling external devices, the main focus of these *restorative* approaches is to provide contingent feedback of specific neuronal states, thereby selectively inducing use-dependent neuroplasticity to normalize pathological brain activity and achieve behavioral gains (Daly and Wolpaw, 2008; Birbaumer et al., 2009). However, affected patients—and even healthy subjects—often show a large variability, or even inability of brain self-regulation, referred to as BCI illiteracy (Vidaurre and Blankertz, 2010). This condition is often related to a low signal-to-noise ratio of the targeted brain activity caused by either physiological (e.g., the depth of the signal source in EEG-based approaches) or pathological (e.g., loss of neural tissue after stroke) mechanisms, or is a result of a misalignment of the mental strategy used by the subject and the brain states targeted by the classifier.

This misalignment may occur when the subject explores different strategies in the course of BCI training, whereas the classifier is usually trained on the first strategy only. Alternative strategies applied by the subject therefore become insufficient. To address these shortcomings, various machine learning techniques and co-adaptive algorithms have been proposed. These adjust the brain state targeted by the classifier to the strategy switching of the subject so as to maximize the classification accuracy (Vidaurre et al., 2011; Bryan et al., 2013). Such approaches are powerful for *assistive* BCIs which can, for example, detect the subject's intention to move and to operate external devices. However, in these approaches, the classifier adapts (Vidaurre et al., 2011; Bryan et al., 2013), and so the subject has no incentive to achieve specific brain states. These adaptation approaches therefore clash with the goal of *restorative* BCIs to modify neuronal activity via operant conditioning, i.e., to achieve specific brain states regarded as beneficial for motor recovery.

Due to the treatment rationale of modulating specific brain features, the classifier of restorative BCIs is usually constrained. In the case of motor rehabilitation, for example, the feature space might be restricted to event-related spectral perturbation in the β-range (Gharabaghi et al., 2014). Moreover, event-related desynchronization has been shown to reflect the excitability of the corticospinal system (Takemi et al., 2013). This interaction between a constrained classifier and the subject, who should be rewarded for achieving specific brain states, poses a special challenge for the optimization of neurofeedback in restorative BCI approaches. Thus, classifier adaptation might affect the treatment rationale of the intervention. In this context, threshold adaptation might be an alternative approach for restorative interventions.

However, we have no theoretical or empirical knowledge as to how threshold adaptation during an intervention might affect reinforcement learning. In restorative BCIs, classifiers are often based on linear discriminant analysis (Theodoridis and Koutroumbas, 2009), e.g., automatic feature weighting based on common spatial patterns (Ang et al., 2014) or the visual inspection and selection of spatially weighted frequency bands (Ramos-Murguialday et al., 2013). These linear methods are characterized by threshold selection, i.e., the definition of a specific value on a one-dimensional continuum spanned between the two states that are to be differentiated. Changing this threshold will modify the sensitivity and the specificity of the classifier regardless of the feature weights (Thompson et al., 2013). The selection of this threshold is currently determined by the intent to maximize the classification accuracy (Thomas et al., 2013; Thompson et al., 2013). Furthermore, the magnitude of classification accuracy is usually perceived as the measure to determine the subject's ability to perform the neurofeedback task (Blankertz et al., 2010; Hammer et al., 2012).

Within the framework of communication theory, a high classification accuracy pertains to a good signal-to-noise ratio of the feedback, i.e., it represents sufficient specificity and sensitivity of the feedback (Thompson et al., 2013). Since there is evidence that erroneous feedback affects the reward system (Balconi and Crivelli, 2010), training at the threshold which results in maximum classification accuracy might be considered as the optimal instructional efficacy.

However, to date, no theoretical or empirical work is available on the relationship between instructional efficacy, threshold adaptation and classification accuracy. We therefore present a theoretical framework for adaptive approaches in restorative BCIs. More specifically, we analyzed how classification accuracy is related to instructional efficacy and whether this instructional efficacy can be improved by threshold adaptation. This research question is related to three components: (1) The theoretical framework to model a neurofeedback environment. (2) The simulation of neurofeedback learning. (3) Adequate measures for instructional efficacy.

On the psychological level, neurofeedback training is aptly described as reinforcement learning (Sherlin et al., 2011). Several mathematical algorithms, most of which were developed as machine learning algorithms (Sutton, 1998; Strens, 2000; Szepesvári, 2010) are now available for reinforcement learning. For various reasons, the simulation of reinforcement learning in the present study is based on a Bayesian algorithm (Strens, 2000). There is ample evidence that sensorimotor integration and learning can be appropriately simulated with a Bayesian model (Körding and Wolpert, 2004; Tin and Poon, 2005; Genewein and Braun, 2012). Bayesian reinforcement learning includes an implicit balancing of exploitation and exploration without the need for additional parameters (Strens, 2000). It has also been proposed as an optimal calculus for defining the rational action selection of human agents (Jacobs and Kruschke, 2011). We therefore developed a Bayesian reinforcement learning model for restorative brain-computer interfaces, and explored the predictions of this model for different threshold adaptation strategies and classification accuracies.

## Mathematical model of the neurofeedback environment

The basic element of any neurofeedback learning environment is that the subject is in a specific state (s), selects one of two possible actions (a), and is rewarded on the basis of the state (s') resulting from this action selection. The training action (a_T_) places the subjects into the training state (s_T_), which is supposed to be rewarded, and (a_F_) places the subjects into the false state (s_F_), which is not supposed to be rewarded.

In any neurofeedback task, the subject can select either the false action (a_F_) (e.g., rest or insufficient neuromodulation), or the trained action (a_T_) (i.e., sufficient neuromodulation). In an ideal neurofeedback intervention, the therapist has perfect knowledge about the current state of the subject and can reward accordingly. In a practical neurofeedback intervention, the subject's current state is determined with only limited specificity and sensitivity, resulting in the possibility of reward for both the trained action P(r|a_T_) and the false action P(r|a_F_).

**Figure 1:**
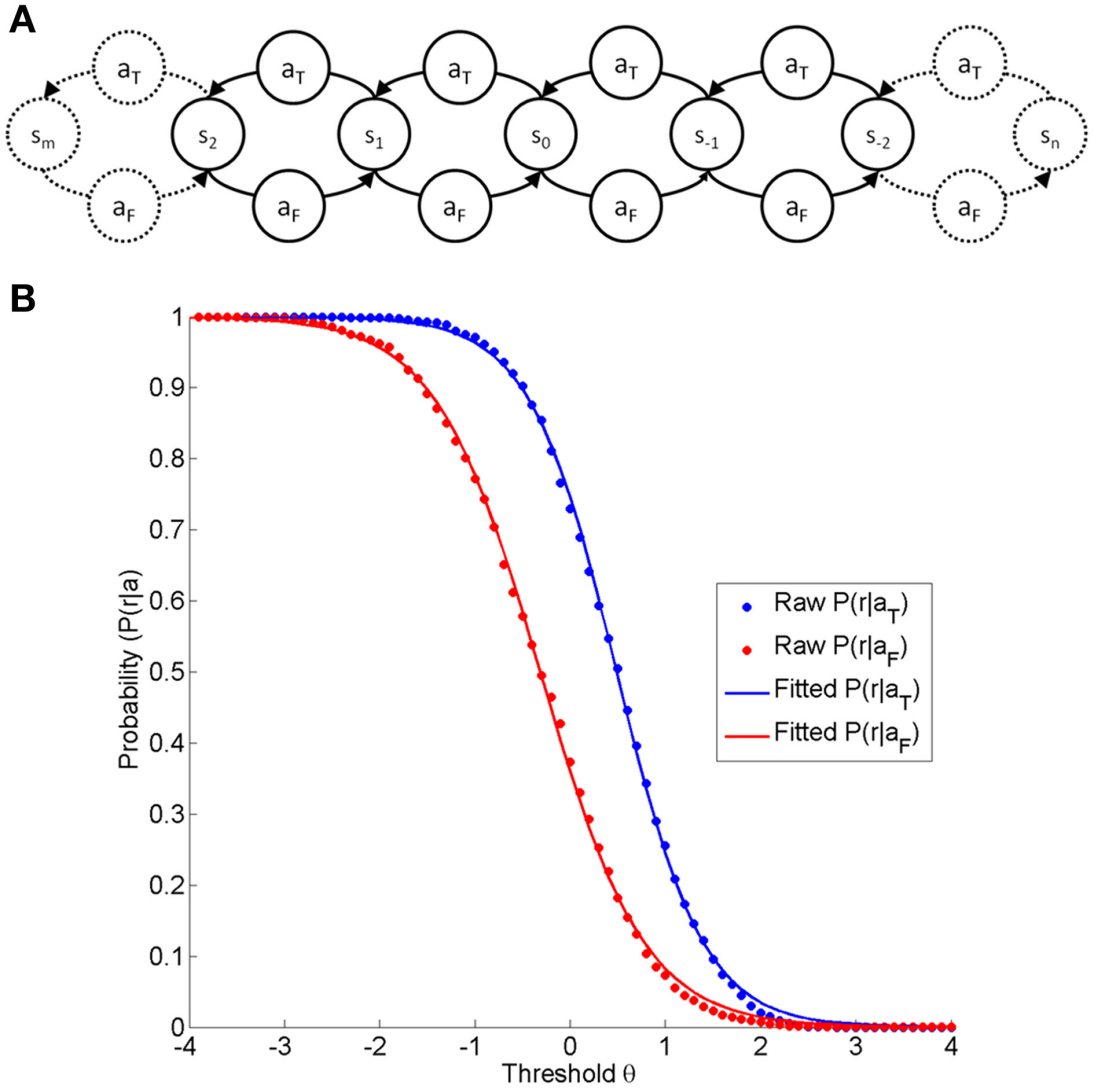
**(A)** is a depiction of the state-action-element fundamental to any neurofeedback environment on the basis of linear discrimination. At any states, the subject selects one of two actions (a_F_, a_T_), resulting in a subsequent state step in the opposite direction (a_F_:false action; a_T_:trained action). **(B)** Shows the probability of reward for a given action (blue a_T_ and red a_F_) as a function of the threshold θ. The dot markers indicate the reward probabilities at different thresholds acquired from a real dataset (a right-handed female subject performing a neurofeedback task based on motor imagery-related β-modulation over sensorimotor regions with contingent haptic feedback, identical to the task described elsewhere, Vukelić et al., 2014). The red and blue traces are logistic functions fitted to the raw data.

In addition, the state space is usually not discrete, but continuous. By including a parameter (δ) for the step size of one action, a continuous state space can be modeled. Assuming that the step size for both actions is equal but that it is taken in different directions, the current state position (σ) in this continuum can be calculated as the number of times the trained action is chosen instead of the false action, i.e., σ = nδ-mδ. The trained action moves the subject one step toward the trained state, whereas the false action moves the subject one step toward the false state (see Figure 1A). This enables us to set a threshold (θ) in the state continuum to determine the probability of reward for the trained action P(r|a_T_) and for the false action P(r|a_F_).

In any neurofeedback environment, the classification at each threshold will therefore result in particular probabilities for reward, thus leading to the characteristic curve shape (see Figure 1B). At each point defined by state (σ) and threshold (θ), the reward rate will adhere to a binomial distribution. The shape across the threshold/state dimension can be adequately modeled by a logistic function (see Figure 1B), which is defined by the discriminatory steepness (D) and the relative position, i.e. the distance (Δ) between the two functions.

$$\begin{array}{l} \hat{P}\left(r\;|\;a_{T};\;\theta ,\;\Delta ,\;\sigma \right)\;=\;\frac{1}{1+e^{D(\theta -\Delta +\sigma )}}\\ \hat{P}\left(r\;|\;a_{F};\;\theta ,\;\Delta ,\;\sigma \right)\;=\;\frac{1}{1+e^{D(\theta +\Delta +\sigma )}}\\ \;\sigma \;=\;\left(n-m\right)\delta \end{array}$$

We therefore postulate that any neurofeedback task based on linear discrimination is fully described by the subject's position in a continuous state space σ, i.e., the history of selected actions n and m, the subject's step size δ, the threshold θ set by the instructor, the classifier steepness D and the distance Δ between the reward probabilities with *D*, Δ ∈ ℝ^≥ 0^ and θ, σ ∈ ℝ and *n*, *m* ∈ ℕ_0_. This function returns symmetric curves, with the shape depending on D only, and the location of each curve depending on Δ and δ.

The parameters σ, θ, and Δ, δ are in arbitrary units and point in the same dimension. We propose that D and Δ are determined by the features selected for the classifier, in particular their signal-to-noise rate and their relative weight. Regardless of these two parameters, the probability of reward for each action is a result of the threshold θ, which is set by the instructor, and the state position σ, which is the result of the subject's history of selected actions and the ability to switch between states, i.e., the step size δ. In this respect, δ and Δ define the shape of classification accuracy across the θ/σ dimension. On account of this common influence, the classification accuracy has ambiguously been interpreted as indicating not only the classifier performance (Thompson et al., 2013) but also the subject's ability (Blankertz et al., 2010; Vidaurre and Blankertz, 2010). However, Δ is determined by the classifier and δ is determined by the subject. By altering the environmental parameter's discrimination D, step size δ and distance Δ, this parametric model enables us to model specific neurofeedback environments. The hatted $\hat{\text{P}}$ indicates that the shape of the reward probability function remains fixed by retaining the discrimination D, the step size δ and the distance Δ constant within the model. It should be noted that, for a fixed environment $\hat{P}$, the distribution of reward for any of the two actions is fully defined by the threshold θ and the state σ.

## Mathematical model of neurofeedback learning

By setting the threshold θ, the instructor may therefore influence the probability distribution of reward for both the trained action P(r|a_T_) and the false action P(r|a_T_), even without direct knowledge about P(a_T_) and P(a_F_). The subject controls P(a_T_) and P(a_F_), although he/she has no direct knowledge about P(r|a_T_) and P(r|a_F_). As a rational agent, the subject will attempt to increase P(r), i.e., exploring and exploiting the most rewarding action, on the basis of the knowledge about the reward probability distribution gained from earlier attempts (Ortega and Braun, 2010a). This can be simulated with a Bayesian reinforcement learning model (Strens, 2000). Within this framework, the probability of reward for each action is a binomial distribution that is perceived by the subject as a beta distribution. The beta distribution is a conjugate prior for the binomial distribution. Like the binomial distribution, the beta distribution describes a continuous probability distribution in the interval [0,1]. In addition, it is controlled by the parameters α and β, which allow modeling of the subject's belief P′ about the true reward probabilities P.

$$\begin{array}{l} P^{\prime }\;\left(r\;|\;a_{T}\right)\sim Beta(\alpha_{T},\beta_{T})\\ P^{\prime }\left(r\;|\;a_{F}\right)\sim Beta(\alpha_{F},\beta_{F}) \end{array}$$

In practical terms, the anticipated reward r_T_ and r_F_ for each action is determined by relative values of α and β, while the confidence of the subject that the anticipated value is true will be determined by the magnitude of α and β. For the novice subject, the beta distributions parameters about the false and true reward (α_F_,α_T_,β_F_,β_T_) are set to 1, and the belief is therefore a uniform distribution.

$$\begin{array}{l} r_{T}=\frac{\alpha_{T}}{\alpha_{T}+\beta_{T}}\\ r_{F}=\frac{\alpha_{F}}{\alpha_{F}+\beta_{F}} \end{array}$$

Since the instructor has only limited knowledge about the action performed by the subject, i.e., the specificity and the sensitivity of the classifier are not perfect, the magnitude of reward has to be identical for a_T_ and a_F_, and only their probabilities differ. By way of a practical example: a robotic orthosis extending the hand of a stroke patient contingent with specific brain states would provide the same haptic/proprioceptive feedback regardless of whether the control signal is achieved by motor imagery-related brain modulation (the intended neurofeedback training) or by neck muscle artifacts projecting to the scalp (Gharabaghi et al., 2014). The false and the trained action will thus result in rewards of identical quality, but with different probability. This is important because it allows us to run the simulation without any scaling factor for reward (Ortega and Braun, 2010b). The subject's reward belief is therefore sufficiently represented by the belief about the reward probabilities.

In each learning iteration, the subject selects an action on the basis of a higher probability of reward than the alternative action. This can be calculated since the subject's confidence that the reward for an action is higher than a certain value x is given by the cumulative Beta distribution function defined by the action parameters α and β.

$$F\left(x;\;\alpha ,\;\beta \right)=\frac{Beta(x;\;\alpha ,\;\beta )}{Beta(\alpha ,\;\beta )}$$

By comparing the relative confidence of both actions, the probability for each action to be selected can be calculated as follows:

$$\begin{array}{l} P\left(a_{T}\right)=\frac{F\left(r_{F};\;\alpha_{T},\;\beta_{T}\right)}{F\left(r_{T};\;\alpha_{F},\;\beta_{F}\right)+F\left(r_{F};\;\alpha_{T},\;\beta_{T}\right)}\\ P\left(a_{F}\right)=\frac{F\left(r_{T};\;\alpha_{F},\;\beta_{F}\right)}{F\left(r_{T};\;\alpha_{F},\;\beta_{F}\right)+F\left(r_{F};\;\alpha_{T},\;\beta_{T}\right)} \end{array}$$

In practical terms, if the subject has little confidence that one action is more likely to return a reward than the other action, both actions will be performed with the same probability, i.e., P(a_T_) equals P(a_F_). If the subject is very confident that a_T_ is more likely to return a reward than a_F_, a_T_ will be more probable, whereas, in the limiting case, P(a_T_) and P(a_F_) would equal one and zero, respectively. Learning in a neurofeedback environment is therefore modulated by the subject's beliefs and confidence about the probability for reward by each action.

In each learning iteration, the action is selected at random on the basis of the subjects belief and confidence in the reward probability (Thompson, 1933; Ortega and Braun, 2010a). The state position σ is subsequently updated by taking a step of the size δ in the chosen direction (false action n + 1, trained action m + 1). Depending on the threshold θ set by the instructor within the otherwise fixed environment $\hat{P}$, a binomial distribution defines the probability for reward. Sampling from this distribution determines whether the action is rewarded (α + 1) or not (β + 1), and the subject will subsequently adjust his/her belief. Afterwards, the next learning iteration begins. Please note that, in this framework, every iteration has an undefined duration. Later in the discussion section, we will reveal how a learning iteration can be understood in a practical application.

### Computational approach

The mathematical model presented here would enable us to estimate the anticipated course of learning for different environments and thresholds by a Monte-Carlo simulation. In this study, we were particularly interested in the anticipated course of learning. Directly increasing the parameters of the Beta distribution by the expectation values for the updates is computationally more efficient than a full computational simulation followed by an averaging across simulations. During each learning iteration, the parameters determining the subject's belief and the state position were therefore updated according to the following formulae:

$$\begin{array}{l} \sigma_{i+1}=\left(n_{i}-m_{i}\right)\delta =\sigma_{i}+E\left[P\left(a_{Ti}\right)-P\left(a_{Fi}\right)\right]\delta \\ \alpha_{i+1}=\alpha_{i}+E\left[P\left(a_{i}\right)\hat{P}\left(r|a,\;\theta ,\;\Delta ,\;\sigma_{i}\right)\right]\\ \beta_{i+1}=\beta_{i}+\left(1-E\left[P\left(a_{i}\right)\hat{P}\left(r|a,\;\theta ,\;\Delta ,\;\sigma_{i}\right)\right]\right) \end{array}$$

Between subsequent learning iterations, the probabilities for reward were updated according to the following formulae:

$$\begin{array}{l} \hat{P}\left(r|a_{T};\;\theta ,\;\Delta ,\;\sigma \right)=\frac{1}{1+e^{D(\theta -\Delta +\sigma )}}\\ \hat{P}\left(r|a_{F};\;\theta ,\;\Delta ,\;\sigma \right)=\frac{1}{1+e^{D(\theta +\Delta +\sigma )}} \end{array}$$

The subject's probability for action selection is of a dynamical nature, as can be readily recognized from these iteratively updated functions.

## Measures of instructional efficiency

The goal of a neurofeedback intervention is to increase the probability of the trained action. As mentioned earlier, this can be affected only by modulating the belief and confidence of the subject about the reward rates for the trained and the false actions, respectively. If the features and thresholds were not adapted, learning would depend on parameters inherent to the subject only, i.e., step size δ. However, the instructor has the option of either adapting the feature weights (affecting D and Δ directly, and σ indirectly) or changing the threshold θ between iterations whenever the environment is fixed (constant D and Δ) due to a certain treatment rationale. In a restorative BCI environment, threshold adaptation will therefore be used to influence the instructional efficiency of the neurofeedback intervention.

However, to explore the predictions of the simulation, objective measures for the instructional efficiency (IE) of the neurofeedback have to be defined. Since the subject's belief and confidence are dynamical, the most straightforward measure would be to take the probability of the trained action for a given threshold θ at each learning iteration i. This would have the advantage of being directly comparable to the optimal learning outcome, which is *P*(*a_t_*) = 1. A further advantage of this approach is that the measure can be translated into entropy with regard to the action selection. This, in turn, can be psychologically interpreted as the subject's uncertainty as to which action is more rewarding. During the course of the training, the subject's uncertainty H should be reduced to zero, and, accordingly, the instructor's goal would also be to reduce the action-entropy to zero. The uncertainty or action entropy H can be calculated as follows:

$$H_{i,\theta }=P\left(a_{T,i},\theta \right)\log_{2}P\left(a_{T,i},\theta \right)+P\left(a_{F,i},\theta \right)\log_{2}P\left(a_{F,i},\theta \right)$$

However, this measure does not divulge whether the subject actually learned in the course of the training, since he/she could have started already with a high probability for the trained action, e.g., if he/she were familiar with the task. This means that the degree to which a subject's uncertainty is reduced might serve as an alternative dynamical measure. Such a measure should consider that a subject's maximum reduction of uncertainty is the difference between the current level of uncertainty and the maximum level of certainty. In accordance with this logic, Georges (1931) defined instructional efficiency as the ratio of the actual gain to the maximum possible gain which can be formulated as follows:

$$IE_{i,\theta }=\frac{P\left(a_{T,i+1},\theta \right)-P\left(a_{T,i},\theta \right)}{1-P\left(a_{T,i},\theta \right)}=\frac{P\left(a_{T,i},\theta \right)di}{P\left(a_{F,i},\theta \right)}$$

Due to the fact that the formula of instructional efficiency IE includes a divisor converging to zero, a singularity will, at some point, occur as lim_*P*(*a_F,i_*,θ)→0_ *IE*_*i*,θ_. This singularity indicates the transition to zero action entropy, and thus the achievement of the training goal.

## Research questions

With these methodical discussions in mind, we now can explore the instructional efficiency of different threshold setting procedures.

### First study

The most frequently used threshold in BCI applications is the one resulting in maximum classification accuracy (Theodoridis and Koutroumbas, 2009).

1. ${\overset{´}{\theta }}_{1}=\arg\max\theta \;\left(P\left(r\;|\;a_{T},\theta \right)\;+P\left(\neg r\;|\;a_{F},\theta \right)\right)$

**Figure 2:**
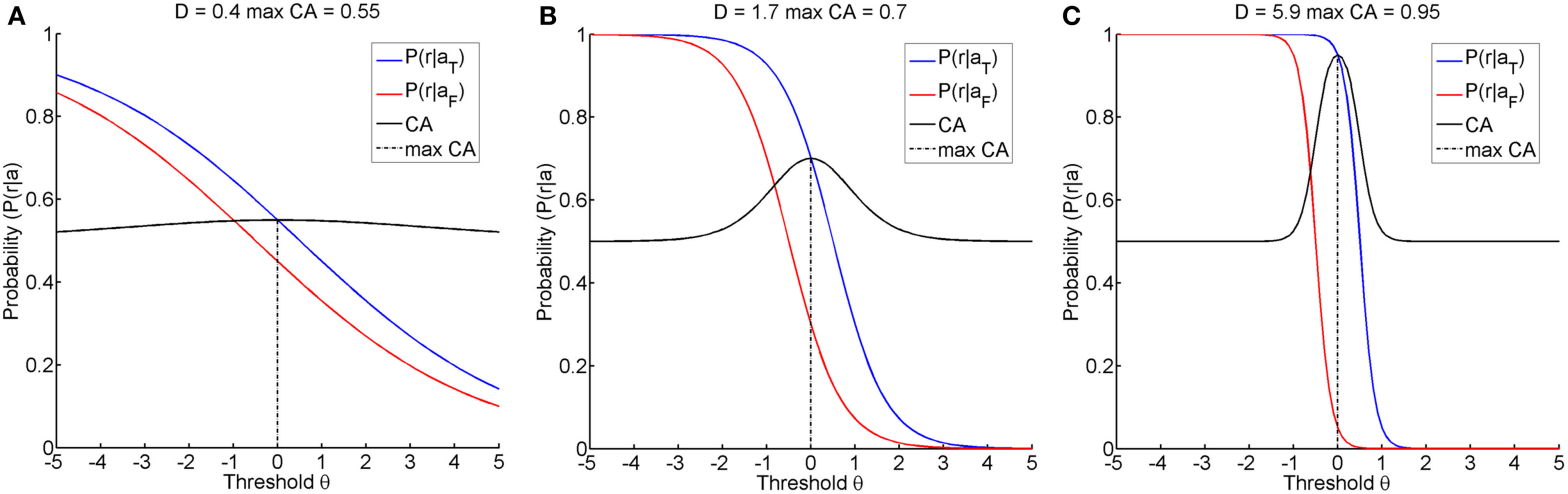
**Shows the three learning environments with different maximum classification accuracies, achieved by selecting an appropriate discriminatory steepness of the model**. **(A)** shows the illiterate environment with low classification accuracy, **(B)** shows the moderate environment with middle classification accuracy, and **(C)** shows the expert environment with high classification accuracy.

The first research goal was to clarify whether instructional efficiency is optimal at this threshold, or whether alternative thresholds might result in a lower action entropy H or in a better instructional efficiency IE. Furthermore, even if the classification accuracy were maximal for a certain threshold, its magnitude could still vary. A classification accuracy of below 70%, for example, has been proposed as an indicator of BCI-illiteracy (Vidaurre and Blankertz, 2010). Furthermore, accuracies close to chance level and close to perfect classification are of particular interest when seeking to improve restorative BCIs. We therefore simulated different classification accuracies, i.e., 55, 70, and 95%, by using a fixed distance Δ of 1 and setting the discriminatory steepness value D to 0.4, 1.7, or 5.9, respectively. We termed these the illiterate, moderate and expert environments accordingly (see Figure 2).

### Second study

We went on to hypothesize that threshold adaptation, i.e., purposefully changing the threshold between iterations, improves the instructional efficiency (IE) and results in lower action entropy (H). To explore the effect of adaptive threshold-setting, we first determined which thresholds resulted in minimal action entropy and maximal instructional efficiency at each iteration across a range of thresholds. Then, instead of using fixed thresholds, we applied the resulting vector as a reference table for the simulation.

1. ${\vec{\theta }}_{i,1}=\arg\min\theta \left({\vec{H}}_{i,\theta }\right)$
2. $\;{\vec{\theta }}_{i,2}=\arg\max\theta \left({\vec{IE}}_{i,\theta }\right)$

In practice this meant that, for every iteration, we measured the threshold with the best instructional efficiency respectively lowest action entropy, resulting in two vectors of thresholds. We then repeated the simulation. In these adaptive runs, we used the respective threshold vector instead of the fixed threshold.

### Realization

**Figure 3:**
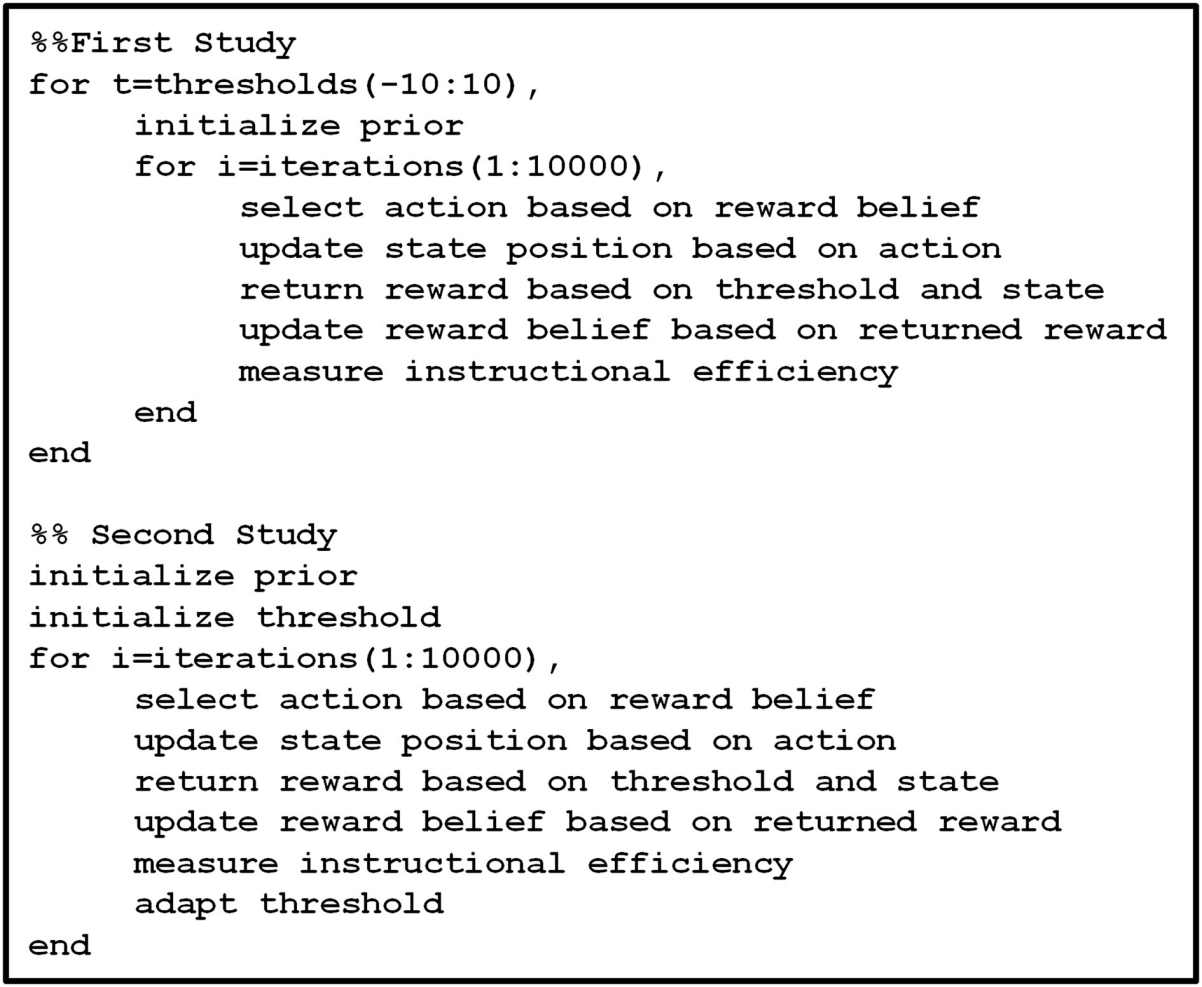
**Shows in pseudo code the computations performed for the reinforcement learning simulation, with the first study exploring the effect of different fixed thresholds, and the second the effect of threshold adaption on the basis of the findings from the first study**.

All simulations were performed for each research question and environment using 10,000 iterations (i), for thresholds (θ) ranging from −10 to 10 and a step size (δ) of 0.1. The prior belief of the subject was initialized by setting α_F_, α_T_, β_F_, and β_T_ to 1. The computations were realized with a custom written code in Matlab R 2014A on a Windows 7 machine. The pseudo-code example (Figure 3) provides a clearer description of this algorithm.

## Results

### Exploration of threshold selection

**Figure 4:**
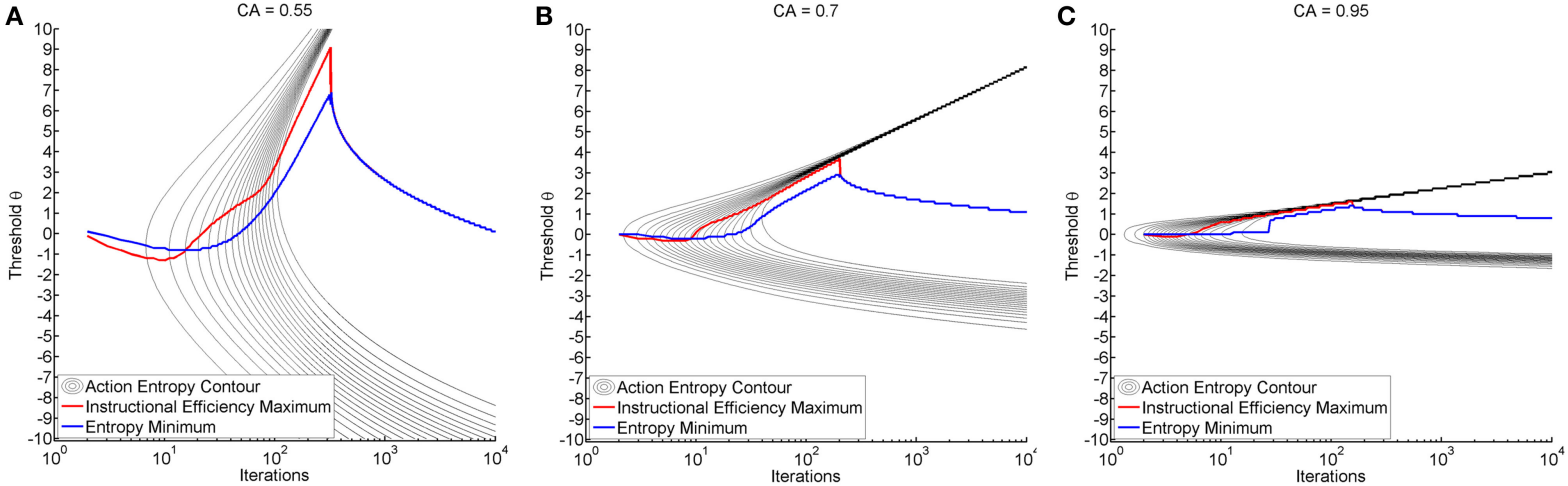
**Shows the time course of action entropy as black contour lines (from 0.95 to 0.05 in steps of 0.05)**. The figures also show the threshold resulting in minimum entropy (blue trace) and maximum instructional efficiency (red trace) for each specific iteration. Training was performed with a fixed threshold (y-axis) and results are shown over iterations (x-axis in logarithmic scale). Subplots depict the illiterate **(A)**, moderate **(B)** and expert **(C)** environment.

We observed a characteristic beam-like shape of progression toward minimal entropy originating from the threshold of maximum classification accuracy (see black trace in Figure 4). In all environments, reduction of entropy first commenced at the threshold of maximum classification accuracy, particularly in environments with higher classification accuracy. Interestingly enough, the range of thresholds that resulted in a reduction of action entropy was narrower for the expert than for the illiterate environment (see Figures 4A–C). Later, the transition between high and low entropy was at higher thresholds than at maximum classification accuracy (CA) thresholds. However, once learning commenced, transition to low entropy was more rapid. This was expressed by a highly asymmetric pattern of entropy reduction (see Figure 4).

It is also worth mentioning that the thresholds which resulted in minimum action entropy and maximum instructional efficiency were not identical to those for maximum classification accuracy and that they varied during the iterations (see Figure 4). The pattern was similar across environments, and was characterized by an early negative and late positive deflection of the action entropy minima (blue trace in Figure 4), which occurred earlier and more steeply for the instructional efficiency maxima (red trace in Figure 4). The negative deflection peaked between iterations 9 and 10 at a threshold of −1.3 for the illiterate environment, between iterations 5 and 8 at a threshold of −0.3 for the moderate environment, and between iterations 3 and 4 at a threshold of −0.1 for the expert environment. The positive deflection peaked between iterations 319 and 322 at a threshold of 9.1 for the illiterate environment, between iterations 198 and 202 at a threshold of 3.7 for the moderate environment, and between iterations 141 and 155 at a threshold of 1.6 for the expert environment. The magnitude of the deflections was therefore higher for low classification accuracy, whereas transitions were faster for higher classification accuracy.

### Exploration of threshold adaptation

**Figure 5:**
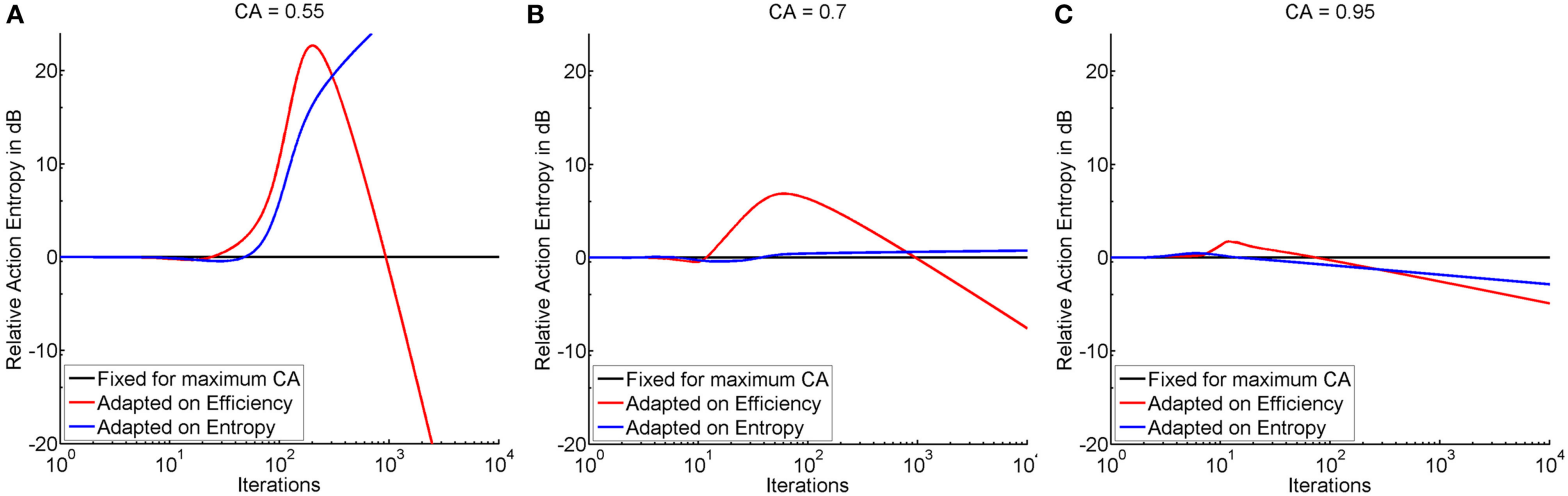
**Shows the time course of action entropy (y-axis in decibel) over iterations (x-axis in logarithmic scale) expressed as action entropy during threshold adaptation on the basis of minimum action entropy (blue trace), and maximum instructional efficiency (red trace) divided by action entropy during training with a fixed threshold at maximum classification accuracy (black trace)**. Subplots show the illiterate **(A)**, moderate **(B)**, and expert **(C)** environment.

Threshold adaptation was performed either following the vector of thresholds that resulted in maximum instructional efficiency (see red trace in Figure 4) or minimum action entropy (see blue trace in Figure 4), and compared to a threshold fixed at maximum classification accuracy. The comparison showed that adaptation based on the instructional efficiency resulted in a phase of comparatively higher action entropy during the training. Subsequently, however, the entropy decreased more rapidly and more steeply, as indicated by a crossing of the trace for adaptation (instructional efficiency) with the trace for fixed threshold (see Figure 5). This pattern was most pronounced for the illiterate environment (see Figure 5A), and similar in shape, but with lower magnitude for the other environments (see Figures 5B,C). Interestingly enough, the final relative entropy was also smaller for the illiterate environment (see Figure 5A).

In the illiterate environment, adaptation on the basis of efficiency resulted in higher action entropy, i.e., a less successful performance, between iterations 24 and 931 and in lower action entropy, i.e., a better performance, thereafter. Adaptation based on entropy was less successful than training with a fixed threshold between iterations 3 and 4 and from 48 onwards (see Figure 5A). In the same vein, adaptation based on entropy was not as good in the moderate environment as training with a fixed threshold between iterations 3 and 6 and from 37 onwards, whereas adaptation based on efficiency resulted in a poorer performance at iteration 3 and between 12 and 959 and in a better performance thereafter (see Figure 5B). In the expert environment, adaptation based on efficiency result in a poorer performance between iterations 3 and 74 and a better performance thereafter, and adaptation on entropy resulted in a poorer performance between iterations 3 and 15, but in a better performance thereafter (see Figure 5C). In summary, efficiency based adaptation was superior to entropy based adaptation in all conditions, with an initial decrease and a subsequent increase of performance. The magnitude of improvement increased from the expert to the moderate environment and peaked in the illiterate environment. In the moderate and in the illiterate condition, these improvements commenced later, i.e., at ~1000 iterations.

## Discussion

In this study, we developed a model of neurofeedback and reinforcement learning that allows—on a theoretical level—an evaluation of different threshold selection approaches and their potential to optimize neurofeedback in restorative BCIs. We pursued two research questions:

### Dynamic vs. fixed threshold

The first goal was to investigate whether thresholds other than the threshold resulting in maximum classification accuracy would be reasonable within the context of neurofeedback. We observed that learning occurred earliest at the threshold of maximum classification accuracy. However, the pattern of entropy reduction was asymmetric, and we detected a dynamic pattern of early negative and late positive deflection for the thresholds, resulting in maximum instructional efficiency or minimum action entropy (see Figure 4). Our theory is that these two findings (dynamics, asymmetry) indicate that threshold adaptation can be superior to training with any fixed threshold. Furthermore, we ascertained that the magnitude of the deflection is greater for environments with lower classification accuracy. This indicates that the effect of adaptation might be even more pronounced for illiterate than for expert subjects.

### Adaptation might improve reinforcement learning

Our second research goal addressed the question as to whether adaptation can theoretically improve the efficiency of the intervention. To answer this question, we used the threshold vectors resulting in maximum instructional efficiency and minimum action entropy derived from the first study, and applied them dynamically during a second training. For this analysis, we used the time course of action entropy as an outcome measure (see Figure 5). We ascertained that threshold adaptation based on action entropy was worse than training with a fixed threshold. By contrast, adaptation for instructional efficiency caused a delayed onset of action entropy reduction, but with a subsequently steeper slope, thus resulting in a stronger and faster overall decrease.

Due to this finding, we consider threshold adaptation as potentially superior to training with a fixed threshold. This effect was especially pronounced for the BCI illiterate condition. We also discovered that the late deflection was strongest in this condition. Since a strong deflection leads to a reduced reward rate, this result indicates that subjects can maintain a low action entropy, even under conditions of reduced reward. This is indicative of successful operant conditioning which is resistant to extinction when reinforcement is lacking. This might be an important asset with regard to the long-term clinical efficacy of restorative BCIs.

### Asymmetric divergence of reward probability

Furthermore, our first study suggests that the effect of adaptation is linked to the transition from negative to positive deflection and to the asymmetry of learning across different thresholds (see Figure 4). Such asymmetry might be relevant for a number of reasons. The probability of reward is the information that is essential to the subject if he/she is to learn which action is more rewarding (Ortega and Braun, 2010b). The distance between the reward probability distribution for the trained and the false action therefore constitutes the most important piece of information for the subject with regard to the question as to which action is better. While classification accuracy is symmetric, measures for the distance of two distributions usually are not, as indicated by the Kullback-Leibler divergence that can be calculated as follows:

1. $KL\left(P\left(r\;|\;a_{T},\theta \right),P\left(r\;|\;a_{F},\theta \right)\right)=P\left(r\;|\;a_{T},\theta \right)\log_{2}\frac{P\left(r\;|\;a_{T},\theta \right)}{P\left(r\;|\;a_{F},\theta \right)}$
2. $KL\left(P\left(r\;|\;a_{F},\theta \right),P\left(r\;|\;a_{T},\theta \right)\right)=P\left(r\;|\;a_{F},\theta \right)\log_{2}\frac{P\left(r\;|\;a_{F},\theta \right)}{P\left(r\;|\;a_{T},\theta \right)}$

**Figure 6:**
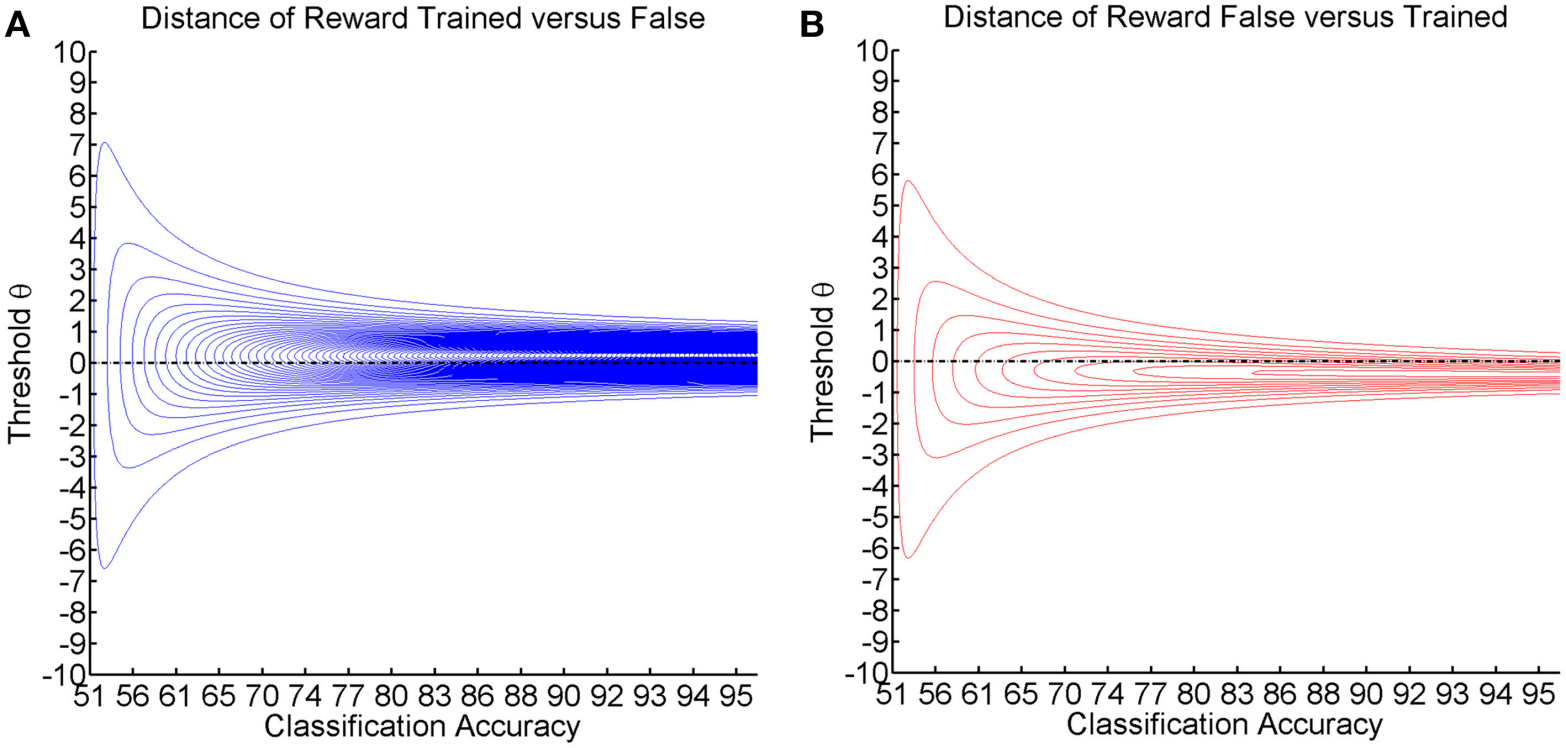
**Shows the visualization of the point-wise Kullback-Leibler divergence between the probability of reward for the trained/false actions, with threshold θ on the y-axis and classification accuracy on the x-axis**. Red contour lines indicate negative values and blue lines positive values (lines have a distance of 0.05). The black line depicts the threshold resulting in maximum classification accuracy. **(A)** Shows the reward caused by preference of the trained action. **(B)** Shows the loss caused by preference of the false action.

This point-wise Kullback-Leibler divergence for each threshold measures the relative informational content of the reward gained by preferring the trained action (see Figure 6A) or the reward lost by preferring the false action (see Figure 6B). The visualization for different classification accuracies shows that the gain information peaks at positive thresholds (see Figure 6A), while the loss information peaks at negative thresholds (see Figure 6B). As classification accuracy increases, the divergence becomes stronger and narrower without affecting the peak location. We postulate that these two stable peaks explain not only the asymmetry and the decreased magnitude of deflection but also the narrow learning space for the expert environment (see Figure 4). In the same vein, classification accuracy narrows down and assumes a more peaked shape in the expert environment (see Figure 2). This indicates that the classification accuracy encompasses a zone in which learning may occur, while the ideal threshold within this zone would have to be selected dynamically in accordance with the subject's current bias. This perspective would tally with the theory that the classification accuracy is the zone of proximal development (Schnotz and Kürschner, 2007; Bauer and Gharabaghi, 2015).

### Limitation to simulation and linear classification

It should be noted that our study is based on simulated—and not on empirical—data. However, our findings suggest that threshold adaptation is capable of increasing the instructional efficacy of a restorative BCI. Furthermore, we show that threshold adaptation might improve learning, particularly for conditions with low classification accuracy. However, this threshold adaptation is specifically applicable in linear classification approaches. Classification algorithms which are non-linear or which classify in multiple dimensions (Theodoridis and Koutroumbas, 2009) might well show different behavior. Additionally, reinforcement learning might be of less importance for assistive or communication BCIs. In these approaches, the performance of the classifier will probably remain the most important design factor (Thompson et al., 2013). We therefore propose the hypothesis that threshold adaptation is particularly suitable for approaches dealing with linear classification in the constrained feature space of neurofeedback training and restorative BCIs (Vidaurre et al., 2011; Bryan et al., 2013).

### Future applications and validation

The simulation applied in this study is based on the theory of reinforcement learning, meaning that the subject continually updates his/her beliefs about the most rewarding action. Learning iterations are an essential aspect of this conceptual framework. But how do these learning iterations translate into the practical world of neurofeedback training and restorative BCI?

We argue that the duration of a single iteration is not an *absolute* measure such as, for example, one feedback trial or 1 iteration/min of training. Instead, we suggest that it be considered as a *relative* measure of information processing that is performed by the subject in a given training environment. This being the case, every iteration is based on the processing of one unit of reward, while the instructional efficiency of one iteration serves as a measure for the efficiency of one bit of reward to reduce entropy, i.e., to change the belief of the subject toward the training goal (Ortega and Braun, 2010b). Accordingly, the duration of a single iteration may be considered as the time required to communicate one bit of information to the subject and for the information to be processed by the subject. It therefore stands to reason that the bit-rate of restorative BCIs may differ in the same way as the one of assistive/communication BCIs (Thompson et al., 2013). In this context, both quantitative and qualitative influences might affect the bit-rate. Longer interventions might be more effective as they transfer a larger amount of information, resulting in a dosage effect. Moreover, some feedback modalities, such as visual or haptic/proprioceptive feedback, might be more informative than others (Gomez-Rodriguez et al., 2011; Parker et al., 2011). Furthermore, the rate at which information could be processed might be determined by specific traits of the subject, e.g., psychological traits such as cognitive resources (Schnotz and Kürschner, 2007) or physiological and anatomical traits such as the parietofrontal network (Buch et al., 2012; Vukelić et al., 2014). In this respect, both physiological and pathological aspects might limit the capacity of a communication channel. In healthy subjects, for example, the extraneous load caused by distractions or feedback overload from multiple senses might impair information processing (Clark, 2006). In pathological conditions, e.g., following a stroke, patients with impaired afferent pathways (Szameitat et al., 2012) might benefit less from proprioceptive feedback than stroke survivors without this impairment. Furthermore, technological limits, such as the time-resolution of the classifier or the inherent signal-to-noise ratio, may also limit the maximum attainable rate (Sanei, 2007).

On a more positive note, according to our theory, limitations in one domain might be compensated by achievements in another. Such additional measures to increase the learning rate might include the coupling of the neurofeedback training with brain stimulation (Lefebvre et al., 2012; Gharabaghi et al., 2014), the monitoring of cognitive resources and engagement based on physiological measures (Smith et al., 2001; Novak et al., 2010; Koenig et al., 2011; Grosse-Wentrup and Schölkopf, 2012), and/or patient screening for treatment eligibility (Stinear et al., 2012; Bauer et al., 2014).

The model presented here might serve as a theoretical basis to integrate this abundance of research into the framework of Bayesian reinforcement learning. Further research will be required to confirm our predictions. Most importantly, however, these findings serve to stimulate empirical studies to seek alternatives to the “maximum classification accuracy” paradigm and to explore threshold adaptation as a tool for increasing the instructional efficiency of restorative BCIs.

### Conflict of interest statement

The authors declare that the research was conducted in the absence of any commercial or financial relationships that could be construed as a potential conflict of interest.
